# Meloxicam hydro­chloride

**DOI:** 10.1107/S241431462300202X

**Published:** 2023-03-10

**Authors:** Fermin Flores Manuel, Martha Sosa Rivadeneyra, Sylvain Bernès

**Affiliations:** a Facultad de Ciencias Químicas, Benemérita Universidad Autónoma de Puebla, 72570 Puebla, Pue., Mexico; b Instituto de Física, Benemérita Universidad Autónoma de Puebla, 72570 Puebla, Pue., Mexico; University of Antofagasta, Chile

**Keywords:** crystal structure, meloxicam, thia­zole, benzo­thia­zine, polymorphism

## Abstract

The title salt, C_14_H_14_N_3_O_4_S_2_^+^·Cl^−^ [systematic name: 2-(4-hy­droxy-2-methyl-1,1-dioxo-1,2-benzo­thia­zine-3-amido)-5-methyl-1,3-thia­zol-3-ium chloride] is the hydro­chloride derivative of meloxicam, a drug used to treat pain and inflammation in rheumatic disorders and osteoarthritis. Although its mol­ecular structure is similar to that previously reported for the hydro­bromide analogue, both salts are not isomorphous. Different crystal structures originate from a conformational modification, arising from a degree of rotational freedom for the thia­zolium ring in the cations. By taking as a reference the conformation of meloxicam, the thia­zolium ring is twisted by 10.96 and −16.70° in the hydro­chloride and hydro­bromide salts, while the 1,2-benzo­thia­zine core is a rigid scaffold. This behaviour could explain why meloxicam is a polymorphous compound.

## Abstract 2

The crystal structure determined for meloxicam hydro­chloride is not isomorphous with the known structure of the hydro­bromide analogue.

## Graphical abstract

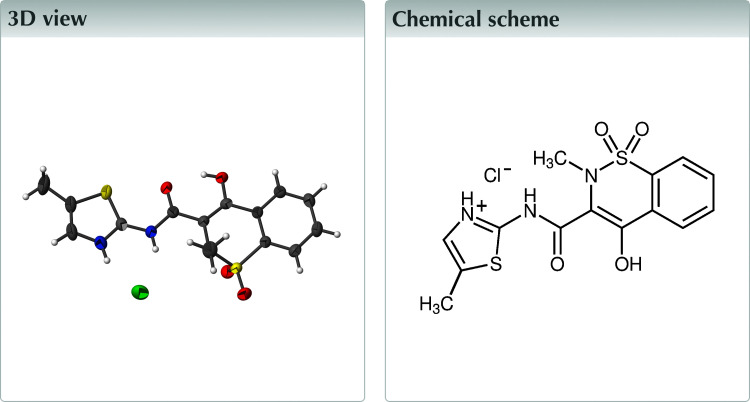

## Structure description

Meloxicam [abbreviated hereafter as MX; systematic name: 4-hy­droxy-2-methyl-*N*-(5-methyl-1,3-thia­zol-2-yl)-2*H*-1,2-benzo­thia­zine-3-carboxamide 1,1-dioxide] is an achiral benzo­thia­zine drug, practically insoluble in water at physiological pH (Luger *et al.*, 1996 ▸). This mol­ecule was patented in 1977, and is currently classified as an anti­pyretic and non-steroidal anti-inflammatory medication, used for the management of pain and inflammation associated with rheumatoid arthritis and osteoarthritis, in adults and children. In some countries, it has also been approved for use in veterinary medicine. The crystallization of meloxicam is a ‘difficult art’ (Śniechowska *et al.*, 2021 ▸), since four neat polymorphic forms are known, along with one hydrated form (Coppi *et al.*, 2003 ▸; Freitas *et al.*, 2017 ▸). So far, only the triclinic form I and the hydrated form were structurally characterized by X-ray diffraction (Luger *et al.*, 1996 ▸; Fabiola *et al.*, 1998 ▸; Fedorov *et al.*, 2019 ▸). Actually, the formula of MX·H_2_O is not well defined: for the reported structure, the water mol­ecule is disordered over two general positions, with occupancies reported as 0.53 (3) and 0.63 (3).

**Figure 1:**
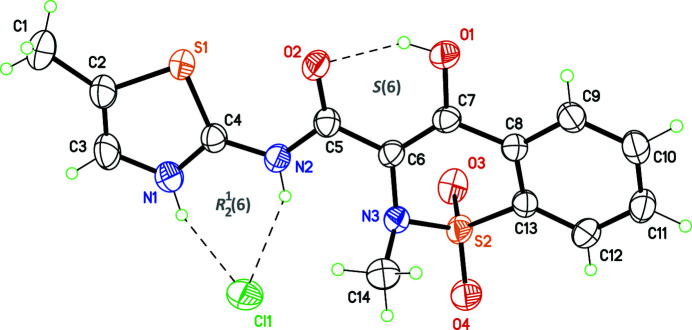
Mol­ecular structure of the title compound, with displacement ellipsoids at the 50% probability level for non-H atoms. Dashed lines represent intra­molecular hydrogen bonds (Table 1 ▸, entries 1–3). The labelling scheme is that adopted for MX·HBr (Tumanov *et al.*, 2011 ▸).

**Figure 2:**
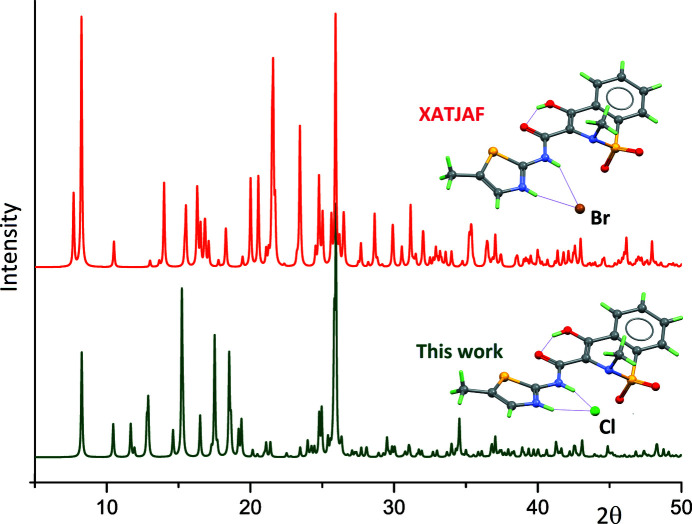
Simulated powder X-ray diffraction patterns for MX·HBr (top, pattern calculated using the deposited Cif file for XATJAF; Tumanov *et al.*, 2011 ▸) and MX·HCl (bottom). Patterns were calculated with *Mercury* (Macrae *et al.*, 2020 ▸), assuming the Cu Kα radiation. Mol­ecular structures are represented along with their patterns.

Among the many meloxicam salts characterized by X-ray diffraction, the hydro­bromide was deposited as a CSD communication (Tumanov *et al.*, 2011 ▸; CSD refcode: XATJAF). MX·HBr crystallizes in space group *P*2_1_/*c*. The thia­zole group is protonated, in such a way that a double-acceptor hydrogen bond is formed with the bromide ion accepting links from the thiazolium and amide NH groups, to form a common 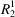 (6) ring motif with the thia­zolium and amide NH groups as donors. The conformation for HMX^+^ is close to that observed for neutral MX, owing to an intra­molecular hydrogen bond between the enol group in the 1,2-benzo­thia­zine core and the carbonyl group of the amide functionality, which gives the common *S*(6) motif. We have now determined the structure of the hydro­chloride salt, MX·HCl, which also crystallizes in space group *P*2_1_/*c*, although with different unit-cell parameters. The mol­ecular structure of MX·HCl is similar to that of MX·HBr, including the same intramolecular O—H⋯O and intermolecular N—H⋯Cl hydrogen bonds (Fig. 1 ▸; Table 1 ▸, entries 1–3). Mol­ecules are however packed in different ways in both salts, as corroborated by their simulated powder diffraction patterns, which are clearly different (Fig. 2 ▸). If the nature of the anion, Cl^−^ or Br^−^, is not taken into account, MX·HBr and MX·HCl can thus be described as polymorphic forms crystallizing in a single space group.

**Figure 3:**
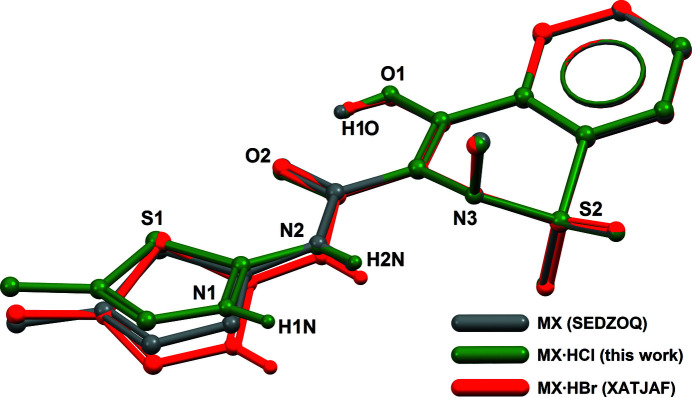
Overlay between meloxicam (grey), MX·HBr (red) and MX·HCl (green), calculated with *Mercury* (Macrae *et al.*, 2020 ▸). The fits were carried out using atoms belonging to the 1,2-benzo­thia­zine core (14 atoms), while the amide and thia­zole groups were kept free. The r.m.s. deviations for the fits are better than 0.04 Å. For the structure of the neutral mol­ecule MX, which has been reported three times, refcode SEDZOQ was retained (Fabiola *et al.*, 1998 ▸), in order to have all models at room temperature. For clarity, halide anions are omitted, as well as H atoms bonded to C atoms. Note that in MX, the thia­zole ring is not protonated.

A close examination of the conformation of the cations, and a comparison with the neutral mol­ecule MX (Fabiola *et al.*, 1998 ▸; CSD refcode: SEDZOQ) rationalizes this behaviour. Assuming that the 1,2-benzo­thia­zine core is a rigid moiety, an overlay between HMX^+^ in both salts and MX shows that the thia­zolium ring has some degree of rotational freedom. Taking MX as reference, the HMX^+^ cation has its thia­zolium ring twisted by 10.96° in MX·HCl and by −16.70° in MX·HBr (Fig. 3 ▸). This rotation over a range of *ca* 25° is sufficient to enable the formation of distinct secondary inter­molecular contacts (Table 1 ▸, entries 4 and 5), which, in turn, alter the packing of the cations in the crystal. By widening this behaviour to meloxicam, for which the rotation of the thia­zole group is less restrained, since no 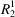 (6) ring motif involving an halide ion is present, one would assume that the rich polymorphism observed for this drug is also associated to similar conformational modifications.

## Synthesis and crystallization

Meloxicam hydro­chloride was unintentionally crystallized while screening slurry co-crystallizations using derivatives of (*S*)-α-methyl­benzyl­amine or l-proline as coformers. In some experiments, an amount of a 0.02 *N* HCl solution was added to the slurry, for the purpose of modifying the pH of the medium. Single crystals of the MX·HCl salt were recovered from these slurries.

## Refinement

Crystal data, data collection and structure refinement details are summarized in Table 2 ▸.

## Supplementary Material

Crystal structure: contains datablock(s) I, global. DOI: 10.1107/S241431462300202X/bx4023sup1.cif

Structure factors: contains datablock(s) I. DOI: 10.1107/S241431462300202X/bx4023Isup2.hkl

Supporting information file. DOI: 10.1107/S241431462300202X/bx4023Isup3.cml

CCDC reference: 2246003

Additional supporting information:  crystallographic information; 3D view; checkCIF report

## Figures and Tables

**Table 1:** Hydrogen-bond geometry (Å, °)

*D*—H⋯*A*	*D*—H	H⋯*A*	*D*⋯*A*	*D*—H⋯*A*
O1—H1*O*⋯O2	0.84 (2)	1.85 (2)	2.5921 (18)	148 (2)
N1—H1*N*⋯Cl1	0.89 (2)	2.16 (2)	2.992 (2)	155.5 (19)
N2—H2*N*⋯Cl1	0.87 (2)	2.35 (2)	3.1185 (18)	147.8 (18)
C1—H1*D*⋯O4^i^	0.96	2.62	3.286 (3)	127
C14—H14*C*⋯O2^ii^	0.96	2.52	3.380 (3)	150

Symmetry codes: (i) 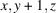 ; (ii) 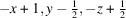 .

**Table 2:** Experimental details

Crystal data	
Chemical formula	C_14_H_14_N_3_O_4_S_2_^+^·Cl^−^
*M*_r_	387.85
Crystal system, space group	Monoclinic, *P*2_1_/*c*
Temperature (K)	295
*a*, *b*, *c* (Å)	11.3380 (6), 10.7346 (5), 14.5503 (10)
β (°)	109.430 (5)
*V* (Å^3^)	1670.05 (17)
*Z*	4
Radiation type	Ag *K*α, λ = 0.56083 Å
μ (mm^−1^)	0.26
Crystal size (mm)	0.23 × 0.09 × 0.07

Data collection	
Diffractometer	Stoe Stadivari
Absorption correction	Multi-scan *X-AREA* 1.88 (Stoe & Cie, 2019 ▸)
*T*_min_, *T*_max_	0.407, 1.000
No. of measured, independent and observed [*I* > 2σ(*I*)] reflections	40949, 3902, 2285
*R*_int_	0.088
(sin θ/λ)_max_ (Å^−1^)	0.653

Refinement	
*R*[*F* ^2^ > 2σ(*F* ^2^)], *wR*(*F* ^2^), *S*	0.032, 0.074, 0.83
No. of reflections	3902
No. of parameters	234
H-atom treatment	H atoms treated by a mixture of independent and constrained refinement
Δρ_max_, Δρ_min_ (e Å^−3^)	0.26, −0.24

Computer programs: *X-AREA* 1.88 (Stoe & Cie, 2019 ▸), *SHELXT2018/2* (Sheldrick, 2015*a* ▸), *SHELXL2018/3* (Sheldrick, 2015*b* ▸), *XP* in *SHELXTL-Plus* (Sheldrick, 2008 ▸), *Mercury* (Macrae *et al.*, 2020 ▸) and *publCIF* (Westrip, 2010 ▸).

## full crystallographic data

### Crystal data

**Table:**

C_14_H_14_N_3_O_4_S_2_^+^·Cl^−^	*F*(000) = 800
*M**_r_* = 387.85	*D*_x_ = 1.543 Mg m^−^^3^
Monoclinic, *P*2_1_/*c*	Ag *K*α radiation, λ = 0.56083 Å
*a* = 11.3380 (6) Å	Cell parameters from 19546 reflections
*b* = 10.7346 (5) Å	θ = 2.2–26.5°
*c* = 14.5503 (10) Å	µ = 0.26 mm^−^^1^
β = 109.430 (5)°	*T* = 295 K
*V* = 1670.05 (17) Å^3^	Block, yellow
*Z* = 4	0.23 × 0.09 × 0.07 mm

### Data collection

**Table:**

Stoe Stadivari diffractometer	3902 independent reflections
Radiation source: Sealed X-ray tube, Axo Astix-f Microfocus source	2285 reflections with *I* > 2σ(*I*)
Graded multilayer mirror monochromator	*R*_int_ = 0.088
Detector resolution: 5.81 pixels mm^-1^	θ_max_ = 21.5°, θ_min_ = 2.2°
ω scans	*h* = −14→14
Absorption correction: multi-scan X-AREA 1.88 (Stoe & Cie, 2019)	*k* = −14→14
*T*_min_ = 0.407, *T*_max_ = 1.000	*l* = −19→19
40949 measured reflections	

### Refinement

**Table:**

Refinement on *F*^2^	Primary atom site location: dual
Least-squares matrix: full	Secondary atom site location: difference Fourier map
*R*[*F*^2^ > 2σ(*F*^2^)] = 0.032	Hydrogen site location: mixed
*wR*(*F*^2^) = 0.074	H atoms treated by a mixture of independent and constrained refinement
*S* = 0.83	*w* = 1/[σ^2^(*F*_o_^2^) + (0.0329*P*)^2^] where *P* = (*F*_o_^2^ + 2*F*_c_^2^)/3
3902 reflections	(Δ/σ)_max_ = 0.001
234 parameters	Δρ_max_ = 0.26 e Å^−^^3^
0 restraints	Δρ_min_ = −0.24 e Å^−^^3^
0 constraints	

### Special details

**Table:**

Refinement. All H atoms bonded to heteroatoms (H1O, H1N and H2N) were refined with free coordinates, and remaining H atoms were placed in idealized positions, with C—H bond lengths constrained to 0.96 (methyl groups) or 0.93 Å (aromatic CH). All H atoms were refined with calculated isotropic displacement parameters.

### Fractional atomic coordinates and isotropic or equivalent isotropic displacement parameters (Å^2^)

**Table:**

	*x*	*y*	*z*	*U*_iso_*/*U*_eq_	
Cl1	0.97139 (5)	0.37304 (6)	0.36101 (5)	0.05872 (19)	
S1	0.78018 (5)	0.80065 (5)	0.39329 (4)	0.04065 (15)	
S2	0.63352 (4)	0.19526 (5)	0.43965 (4)	0.03731 (14)	
O1	0.39766 (12)	0.51274 (13)	0.39149 (12)	0.0416 (4)	
H1O	0.441 (2)	0.576 (2)	0.3941 (18)	0.062*	
O2	0.59216 (11)	0.64257 (12)	0.39574 (11)	0.0402 (4)	
O3	0.67314 (12)	0.23387 (14)	0.53969 (11)	0.0463 (4)	
O4	0.68678 (13)	0.08624 (14)	0.41315 (13)	0.0544 (5)	
N1	0.94397 (15)	0.64715 (18)	0.38545 (14)	0.0421 (5)	
H1N	0.9763 (19)	0.572 (2)	0.3827 (16)	0.050*	
N2	0.76883 (14)	0.54411 (16)	0.39712 (14)	0.0355 (4)	
H2N	0.8075 (18)	0.4746 (19)	0.3955 (16)	0.043*	
N3	0.65464 (13)	0.31354 (15)	0.37489 (13)	0.0338 (4)	
C1	0.9370 (2)	0.9923 (2)	0.3668 (2)	0.0715 (9)	
H1B	0.886168	1.017468	0.302491	0.107*	
H1C	1.023451	1.007147	0.375131	0.107*	
H1D	0.913849	1.039311	0.414236	0.107*	
C2	0.91763 (19)	0.8566 (2)	0.37994 (18)	0.0474 (6)	
C3	0.9929 (2)	0.7626 (2)	0.37786 (18)	0.0503 (6)	
H3	1.071320	0.773685	0.371823	0.060*	
C4	0.83125 (16)	0.65175 (18)	0.39320 (15)	0.0336 (5)	
C5	0.64622 (16)	0.54299 (19)	0.39282 (15)	0.0329 (5)	
C6	0.58544 (16)	0.42257 (18)	0.38367 (15)	0.0314 (5)	
C7	0.46600 (16)	0.41332 (18)	0.38478 (15)	0.0315 (5)	
C8	0.40231 (15)	0.29411 (18)	0.38082 (14)	0.0304 (4)	
C9	0.27306 (16)	0.28810 (19)	0.35974 (15)	0.0362 (5)	
H9	0.226126	0.360935	0.350480	0.043*	
C10	0.21526 (17)	0.1742 (2)	0.35270 (16)	0.0399 (5)	
H10	0.129213	0.170995	0.339433	0.048*	
C11	0.28195 (18)	0.0647 (2)	0.36485 (17)	0.0420 (5)	
H11	0.240667	−0.011404	0.357289	0.050*	
C12	0.41100 (18)	0.06833 (19)	0.38842 (16)	0.0384 (5)	
H12	0.457202	−0.004976	0.397669	0.046*	
C13	0.46958 (16)	0.18279 (18)	0.39791 (15)	0.0314 (4)	
C14	0.6498 (2)	0.2839 (2)	0.27407 (17)	0.0482 (6)	
H14A	0.699323	0.211130	0.274864	0.072*	
H14B	0.682318	0.352942	0.247956	0.072*	
H14C	0.564780	0.268629	0.234186	0.072*	

### Atomic displacement parameters (Å^2^)

**Table:**

	*U*^11^	*U*^22^	*U*^33^	*U*^12^	*U*^13^	*U*^23^
Cl1	0.0481 (3)	0.0530 (4)	0.0799 (5)	0.0108 (3)	0.0278 (3)	−0.0043 (4)
S1	0.0437 (3)	0.0326 (3)	0.0513 (4)	−0.0087 (2)	0.0233 (3)	−0.0037 (3)
S2	0.0316 (2)	0.0271 (3)	0.0530 (4)	0.0024 (2)	0.0138 (2)	0.0052 (3)
O1	0.0363 (7)	0.0272 (8)	0.0634 (11)	0.0046 (6)	0.0194 (7)	0.0013 (8)
O2	0.0383 (7)	0.0267 (8)	0.0571 (11)	−0.0007 (6)	0.0179 (7)	0.0027 (7)
O3	0.0430 (7)	0.0456 (9)	0.0426 (10)	−0.0028 (7)	0.0039 (7)	0.0088 (8)
O4	0.0467 (8)	0.0310 (8)	0.0918 (14)	0.0089 (7)	0.0316 (9)	0.0046 (9)
N1	0.0338 (9)	0.0423 (11)	0.0521 (13)	−0.0032 (8)	0.0169 (8)	−0.0026 (10)
N2	0.0326 (8)	0.0290 (9)	0.0457 (12)	−0.0017 (7)	0.0141 (8)	0.0003 (9)
N3	0.0342 (8)	0.0279 (9)	0.0428 (11)	0.0000 (7)	0.0173 (8)	0.0012 (8)
C1	0.0752 (17)	0.0440 (16)	0.110 (3)	−0.0216 (13)	0.0501 (18)	−0.0070 (16)
C2	0.0467 (11)	0.0437 (14)	0.0579 (17)	−0.0157 (11)	0.0254 (12)	−0.0081 (12)
C3	0.0397 (11)	0.0536 (15)	0.0611 (17)	−0.0186 (11)	0.0215 (11)	−0.0060 (13)
C4	0.0311 (9)	0.0348 (12)	0.0344 (13)	−0.0046 (8)	0.0101 (9)	−0.0015 (10)
C5	0.0323 (9)	0.0318 (11)	0.0341 (13)	−0.0008 (9)	0.0104 (9)	0.0034 (10)
C6	0.0315 (9)	0.0251 (10)	0.0378 (13)	0.0008 (8)	0.0116 (9)	0.0023 (9)
C7	0.0341 (9)	0.0270 (10)	0.0333 (13)	0.0031 (8)	0.0111 (9)	0.0021 (9)
C8	0.0316 (9)	0.0291 (11)	0.0320 (12)	−0.0015 (8)	0.0125 (8)	0.0000 (9)
C9	0.0330 (9)	0.0365 (12)	0.0414 (13)	0.0008 (9)	0.0156 (9)	0.0027 (10)
C10	0.0321 (9)	0.0445 (13)	0.0448 (14)	−0.0055 (9)	0.0152 (10)	−0.0001 (11)
C11	0.0443 (11)	0.0367 (13)	0.0475 (15)	−0.0123 (10)	0.0186 (11)	−0.0026 (11)
C12	0.0431 (10)	0.0294 (12)	0.0437 (14)	−0.0011 (9)	0.0156 (10)	0.0015 (10)
C13	0.0305 (8)	0.0298 (11)	0.0350 (12)	−0.0012 (8)	0.0123 (8)	0.0013 (9)
C14	0.0536 (12)	0.0463 (14)	0.0492 (15)	−0.0053 (11)	0.0232 (11)	−0.0096 (12)

### Geometric parameters (Å, º)

**Table:**

S1—C4	1.700 (2)	C1—H1D	0.9600
S1—C2	1.740 (2)	C2—C3	1.328 (3)
S2—O4	1.4275 (15)	C3—H3	0.9300
S2—O3	1.4343 (16)	C5—C6	1.450 (3)
S2—N3	1.6455 (17)	C6—C7	1.363 (2)
S2—C13	1.7580 (17)	C7—C8	1.461 (3)
O1—C7	1.341 (2)	C8—C13	1.395 (3)
O1—H1O	0.84 (2)	C8—C9	1.395 (2)
O2—C5	1.240 (2)	C9—C10	1.375 (3)
N1—C4	1.321 (2)	C9—H9	0.9300
N1—C3	1.377 (3)	C10—C11	1.376 (3)
N1—H1N	0.89 (2)	C10—H10	0.9300
N2—C4	1.366 (2)	C11—C12	1.388 (3)
N2—C5	1.371 (2)	C11—H11	0.9300
N2—H2N	0.87 (2)	C12—C13	1.382 (3)
N3—C6	1.438 (2)	C12—H12	0.9300
N3—C14	1.484 (3)	C14—H14A	0.9600
C1—C2	1.495 (3)	C14—H14B	0.9600
C1—H1B	0.9600	C14—H14C	0.9600
C1—H1C	0.9600		
			
C4—S1—C2	90.37 (10)	O2—C5—C6	123.12 (16)
O4—S2—O3	119.55 (10)	N2—C5—C6	117.12 (17)
O4—S2—N3	108.82 (10)	C7—C6—N3	121.01 (17)
O3—S2—N3	107.58 (9)	C7—C6—C5	120.50 (17)
O4—S2—C13	109.75 (9)	N3—C6—C5	118.49 (15)
O3—S2—C13	108.11 (9)	O1—C7—C6	122.89 (18)
N3—S2—C13	101.50 (9)	O1—C7—C8	114.19 (16)
C7—O1—H1O	107.9 (16)	C6—C7—C8	122.91 (17)
C4—N1—C3	113.59 (19)	C13—C8—C9	118.14 (18)
C4—N1—H1N	117.6 (14)	C13—C8—C7	120.63 (15)
C3—N1—H1N	128.8 (14)	C9—C8—C7	121.23 (17)
C4—N2—C5	122.49 (17)	C10—C9—C8	119.81 (19)
C4—N2—H2N	116.9 (13)	C10—C9—H9	120.1
C5—N2—H2N	120.3 (13)	C8—C9—H9	120.1
C6—N3—C14	114.85 (17)	C9—C10—C11	121.45 (17)
C6—N3—S2	112.92 (13)	C9—C10—H10	119.3
C14—N3—S2	115.87 (14)	C11—C10—H10	119.3
C2—C1—H1B	109.5	C10—C11—C12	119.80 (19)
C2—C1—H1C	109.5	C10—C11—H11	120.1
H1B—C1—H1C	109.5	C12—C11—H11	120.1
C2—C1—H1D	109.5	C13—C12—C11	118.80 (19)
H1B—C1—H1D	109.5	C13—C12—H12	120.6
H1C—C1—H1D	109.5	C11—C12—H12	120.6
C3—C2—C1	127.9 (2)	C12—C13—C8	121.86 (16)
C3—C2—S1	110.25 (17)	C12—C13—S2	121.34 (15)
C1—C2—S1	121.73 (18)	C8—C13—S2	116.68 (14)
C2—C3—N1	113.77 (19)	N3—C14—H14A	109.5
C2—C3—H3	123.1	N3—C14—H14B	109.5
N1—C3—H3	123.1	H14A—C14—H14B	109.5
N1—C4—N2	120.09 (18)	N3—C14—H14C	109.5
N1—C4—S1	112.01 (15)	H14A—C14—H14C	109.5
N2—C4—S1	127.88 (14)	H14B—C14—H14C	109.5
O2—C5—N2	119.75 (18)		
			
O4—S2—N3—C6	169.71 (13)	N2—C5—C6—N3	−3.0 (3)
O3—S2—N3—C6	−59.40 (14)	N3—C6—C7—O1	−179.05 (19)
C13—S2—N3—C6	54.01 (15)	C5—C6—C7—O1	1.9 (3)
O4—S2—N3—C14	34.30 (16)	N3—C6—C7—C8	2.3 (3)
O3—S2—N3—C14	165.18 (13)	C5—C6—C7—C8	−176.80 (19)
C13—S2—N3—C14	−81.41 (15)	O1—C7—C8—C13	−164.08 (19)
C4—S1—C2—C3	−1.0 (2)	C6—C7—C8—C13	14.7 (3)
C4—S1—C2—C1	175.5 (2)	O1—C7—C8—C9	16.1 (3)
C1—C2—C3—N1	−175.5 (2)	C6—C7—C8—C9	−165.1 (2)
S1—C2—C3—N1	0.7 (3)	C13—C8—C9—C10	−2.5 (3)
C4—N1—C3—C2	0.1 (3)	C7—C8—C9—C10	177.4 (2)
C3—N1—C4—N2	177.2 (2)	C8—C9—C10—C11	−0.8 (3)
C3—N1—C4—S1	−0.9 (2)	C9—C10—C11—C12	2.5 (4)
C5—N2—C4—N1	−171.6 (2)	C10—C11—C12—C13	−0.9 (3)
C5—N2—C4—S1	6.2 (3)	C11—C12—C13—C8	−2.5 (3)
C2—S1—C4—N1	1.08 (18)	C11—C12—C13—S2	173.43 (17)
C2—S1—C4—N2	−176.9 (2)	C9—C8—C13—C12	4.2 (3)
C4—N2—C5—O2	−7.8 (3)	C7—C8—C13—C12	−175.7 (2)
C4—N2—C5—C6	171.4 (2)	C9—C8—C13—S2	−171.96 (16)
C14—N3—C6—C7	95.1 (2)	C7—C8—C13—S2	8.2 (3)
S2—N3—C6—C7	−40.8 (2)	O4—S2—C13—C12	29.6 (2)
C14—N3—C6—C5	−85.8 (2)	O3—S2—C13—C12	−102.42 (19)
S2—N3—C6—C5	138.34 (16)	N3—S2—C13—C12	144.58 (18)
O2—C5—C6—C7	−4.7 (3)	O4—S2—C13—C8	−154.31 (16)
N2—C5—C6—C7	176.1 (2)	O3—S2—C13—C8	73.71 (18)
O2—C5—C6—N3	176.19 (19)	N3—S2—C13—C8	−39.29 (18)

### Hydrogen-bond geometry (Å, º)

**Table:**

*D*—H···*A*	*D*—H	H···*A*	*D*···*A*	*D*—H···*A*
O1—H1*O*···O2	0.84 (2)	1.85 (2)	2.5921 (18)	148 (2)
N1—H1*N*···Cl1	0.89 (2)	2.16 (2)	2.992 (2)	155.5 (19)
N2—H2*N*···Cl1	0.87 (2)	2.35 (2)	3.1185 (18)	147.8 (18)
C1—H1*D*···O4^i^	0.96	2.62	3.286 (3)	127
C14—H14*C*···O2^ii^	0.96	2.52	3.380 (3)	150

Symmetry codes: (i) *x*, *y*+1, *z*; (ii) −*x*+1, *y*−1/2, −*z*+1/2.

## References

[bb1] Coppi, L., Bartra Sanmartí, M. & Closa Clavo, M. (2003). US patent 2003/0109701 A1.

[bb2] Fabiola, G. F., Pattabhi, V., Manjunatha, S. G., Rao, G. V. & Nagarajan, K. (1998). *Acta Cryst.* C**54**, 2001–2003.

[bb3] Fedorov, A. Y., Drebushchak, T. N. & Tantardini, C. (2019). *Comput. Theor. Chem.* **1157**, 47–53.

[bb4] Jacon Freitas, J. T., Santos Viana, O. M. M., Bonfilio, R., Doriguetto, A. C. & de Araújo, M. B. (2017). *Eur. J. Pharm. Sci.* **109**, 347–358. 10.1016/j.ejps.2017.08.029 28844846

[bb5] Luger, P., Daneck, K., Engel, W., Trummlitz, G. & Wagner, K. (1996). *Eur. J. Pharm. Sci.* **4**, 175–187.

[bb6] Macrae, C. F., Sovago, I., Cottrell, S. J., Galek, P. T. A., McCabe, P., Pidcock, E., Platings, M., Shields, G. P., Stevens, J. S., Towler, M. & Wood, P. A. (2020). *J. Appl. Cryst.* **53**, 226–235. 10.1107/S1600576719014092 PMC6998782 32047413

[bb7] Sheldrick, G. M. (2008). *Acta Cryst.* A**64**, 112–122. 10.1107/S0108767307043930 18156677

[bb8] Sheldrick, G. M. (2015*a*). *Acta Cryst.* A**71**, 3–8.

[bb9] Sheldrick, G. M. (2015*b*). *Acta Cryst.* C**71**, 3–8.

[bb10] Śniechowska, J., Paluch, P. & Dudek, M. K. (2021). *Acta Cryst.* A**77**, C897.

[bb11] Stoe & Cie (2019). *X-AREA* and *X-RED32*, Stoe & Cie, Darmstadt, Germany.

[bb12] Tumanov, N., Dyakonova, M., Pankrushina, N. & Shahtshneider, T. P. (2011). CSD Communication (refcode XATJAF, CCDC 832082). CCDC, Cambridge, England.

[bb13] Westrip, S. P. (2010). *J. Appl. Cryst.* **43**, 920–925.

